# Brain-derived neurotrophic factor released from blood platelets prevents dendritic atrophy of lesioned adult central nervous system neurons

**DOI:** 10.1093/braincomms/fcad046

**Published:** 2023-03-02

**Authors:** Andrew Want, Xinsheng Nan, Eirini Kokkali, Yves-Alain Barde, James E Morgan

**Affiliations:** School of Optometry and Vision Sciences, Cardiff University, Cardiff CF24 4HQ, UK; School of Bioscience, Cardiff University, Cardiff CF10 3AX, UK; School of Optometry and Vision Sciences, Cardiff University, Cardiff CF24 4HQ, UK; School of Bioscience, Cardiff University, Cardiff CF10 3AX, UK; School of Optometry and Vision Sciences, Cardiff University, Cardiff CF24 4HQ, UK

**Keywords:** BDNF, platelet, dendrite, retinal ganglion cell

## Abstract

In humans and other primates, blood platelets contain high concentrations of brain-derived neurotrophic factor due to the expression of the *BDNF* gene in megakaryocytes. By contrast, mice, typically used to investigate the impact of CNS lesions, have no demonstrable levels of brain-derived neurotrophic factor in platelets, and their megakaryocytes do not transcribe significant levels of the *Bdnf* gene. Here, we explore potential contributions of platelet brain-derived neurotrophic factor with two well-established CNS lesion models, using ‘humanized’ mice engineered to express the *Bdnf* gene under the control of a megakaryocyte-specific promoter. Retinal explants prepared from mice containing brain-derived neurotrophic factor in platelets were labelled using DiOlistics and the dendritic integrity of retinal ganglion cells assessed after 3 days by Sholl analysis. The results were compared with retinas of wild-type animals and with wild-type explants supplemented with saturating concentrations of brain-derived neurotrophic factor or the tropomyosin kinase B antibody agonist, ZEB85. An optic nerve crush was also performed, and the dendrites of retinal ganglion cells similarly assessed 7-day post-injury, comparing the results of mice containing brain-derived neurotrophic factor in platelets with wild-type animals. In mice engineered to contain brain-derived neurotrophic factor in platelets, the mean serum brain-derived neurotrophic factor levels were 25.74 ± 11.36 ng/mL for homozygous and 17.02 ± 6.44 ng/mL for heterozygous mice, close to those determined in primates. Retinal explants from these animals showed robust preservation of dendrite complexity, similar to that seen with wild-type explants incubated with medium supplemented with brain-derived neurotrophic factor or the tropomyosin receptor kinase B antibody agonist, ZEB85. The Sholl areas under curve were 1811 ± 258, 1776 ± 435 and 1763 ± 256 versus 1406 ± 315 in the wild-type control group (*P* ≤ 0.001). Retinal ganglion cell survival based on cell counts was similar in all four groups, showing ∼15% loss. A robust neuroprotective effect was also observed following optic nerve crush when assessing the dendrites of the retinal ganglion cells in the transgenic mouse, with Sholl area under the curve significantly higher compared to wild-type (2667 ± 690 and 1921 ± 392, *P* = 0.026), with no significant difference in the contralateral eye controls. Repeat experiments found no difference in cell survival, with both showing ∼50% loss. These results indicate that platelet brain-derived neurotrophic factor has a strong neuroprotective effect on the dendrite complexity of retinal ganglion cells in both an *ex vivo* and *in vivo* model, suggesting that platelet brain-derived neurotrophic factor is likely to be a significant neuroprotective factor in primates.

## Teaser

Unlike humans, mice lack brain-derived neurotrophic factor in their platelets. Want *et al*. used a transgenic mouse model, engineered to express brain-derived neurotrophic factor in blood platelets and demonstrate significant neuroprotection for lesioned retinal ganglion cells compared with wild-type mice.

## Graphical Abstract

**Graphical Abstract:**
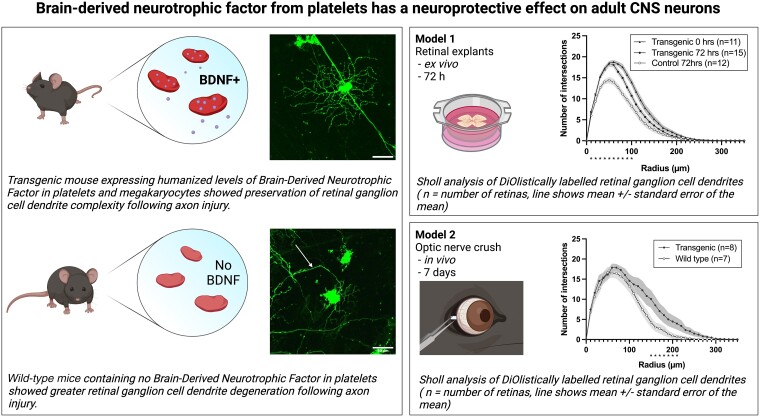

## Introduction

Brain-derived neurotrophic factor (BDNF) is a member of the nerve growth factor (NGF) family widely expressed in the central nervous system (CNS) and peripheral nervous system (PNS). The neuroprotective effects of BDNF have been well-established, in particular its ability to prevent neuronal death during development or after lesion.^1-5^ This early work raised hopes that BDNF could be used as a protective or reparative agent in a variety of neurological conditions. However, the therapeutic application of BDNF has been constrained by its physical properties. It has a high net charge (pI ∼9.6), readily adheres to surfaces such as glass and plastic and diffuses poorly,^6,7^ and when it eventually does enter circulation, it is rapidly filtered by the kidney due to its 26 kD molecular weight. Furthermore, even when BDNF has reached its target receptor, tropomyosin receptor kinase B (TrkB), prolonged exposure causes downregulation of the TrkB receptor and loss of neuroprotective effects.^8,9^ Collectively, these contributing factors have made the successful delivery of exogenous BDNF challenging. A number of strategies have been explored to overcome these technical difficulties, and while some have shown promising results in experimental models,^6,10^ none have been sufficient to support effective clinic translation.

An alternative approach is to explore endogenous, non-neuronal, sources of BDNF that would overcome issues of bioavailability and avoid the need for repeated administration. Platelets are a promising candidate for this physiological method of BDNF delivery since they contain high concentrations of BDNF and account for at least 90% of blood BDNF in humans.^11^ The importance of this store has been highlighted by surprising and hitherto incompletely understood correlations between low serum concentrations of BDNF and conditions such as depression,^12^ Huntington’s disease^13^ and Alzheimer’s disease.^14^ These observations raise the question as to whether platelet BDNF can interact with the CNS despite an apparent inability to cross the blood–brain barrier (BBB) and whether the enhanced release of BDNF from platelet stores could be beneficial in the treatment of neurodegenerative conditions. Unfortunately, the factors that control (CTL) the storage and release of BDNF from platelets are not well-understood, and serum concentrations can show considerable variation under normal conditions.^15^ It is also unclear why BDNF levels in platelets or serum should reflect levels in the brain and what the underlying mechanisms of any association may be, thus highlighting the need for further investigation.

Murine models have been extensively used to investigate mechanisms underlying CNS disorders due to the availability of well-established disease models and in particular of genetically modified animals. However, mice differ from humans and other primates in that their platelets do not contain BDNF and their megakaryocytes do not transcribe the *Bdnf* gene at significant levels, to the extent that BDNF is undetectable in mouse megakaryocytes.^16^ To circumvent this problem, we developed a mouse model using a megakaryocyte-specific promoter excising a stop cassette from a ubiquitously expressed BDNF construct [*Rosa26-LSL-Bdnf-myc-IG/Pf4iCre^+^* (B Cre+) mouse].^17^ These animals express levels of BDNF similar to those found in human platelets, therefore allowing the functional relevance of this novel source of BDNF to be explored. Here, we used two well-established CNS lesion models, retinal explants and optic nerve crush (ONC), to examine and compare the extent of retinal ganglion cell (RGC) damage in engineered versus wild-type (WT) mice. Injury to the optic nerve provides a predictable degeneration of RGCs and can be achieved in *ex vivo* (retinal explant) and *in vivo* (ONC) models with reliable readouts of RGC integrity.^18,19^ RGCs were labelled using DiOlistics and dendritic atrophy, a sensitive measure of RGC health that occurs before cell death,^20^ quantified by Sholl analysis. These results were then supplemented by measurements of cell survival using RGC counts. The main purpose of this study was thus to explore the potential neuroprotective role of platelet-derived BDNF by replicating in the mouse the situation prevailing in humans.

## Materials and methods

### Animal husbandry

All animals were used in compliance with the Home Office Animals (Scientific Procedures) Act, 1986, and with the Association for Research in Vision and Ophthalmology (ARVO) Guidelines for the Use of Animals in Ophthalmic and Vision Research. Mice were housed in M3 cages (48 × 15 × 13 cm, 510 cm^2^ floor space) with one to five adult mice per cage. Mixed genotypes were housed together (i.e. homozygous/heterozygous/WT mice were not separated) at a controlled room temperature of 20–24°C and humidity of 55% ± 10%. They were maintained on a 12-h light/dark cycle with access to food *ad libitum*.

A total of 82 mice were used in the study, 41 Cre+ mice expressing *Bdnf* (mean age 3.95 ± 0.80 months, 23 males and 18 females) and 41 Cre− mice, phenotypically WT (3.66 ± 1.99 months, 24 males and 17 females). There were no significant differences in weights between Cre+ and Cre− mice (for males, Cre+: 27.06 ± 4.51 g, Cre−: 27.22 ± 1.73 g; for females, Cre+: 21.01 ± 1.21 g, Cre−: 21.01 ± 0.55 g).

### Development of the B Cre+ mouse

A tagged version of *Bdnf* (*Bdnf-myc*), followed by an internal ribosome entry site sequence allowing the translation of green fluorescent protein (GFP), was inserted into the Rosa26 locus of the mouse genome. A preceding stop cassette was then excised by crossing with the *PF4 Cre* line (The Jackson Laboratory, 008535, C57BL/6 background), allowing transcription in megakaryocytes (see Dingsdale *et al.*^17^ for details).

## Enzyme-linked immunosorbent assay

For enzyme-linked immunosorbent assay (ELISA) measurements of serum BDNF levels, mice were culled using CO_2_ with blood samples collected immediately after death by cardiac puncture. For serum preparation, whole blood samples were kept at room temperature for 1 h, followed by 4°C for 1 h before being centrifuged at 2000*g* for 10 min at 4°C. The top liquid fraction was collected and centrifuged again using the same parameters to remove any remaining cells. The BDNF concentration was measured using the Mature BDNF ELISA Kit Wako, High Sensitivity, 298-83901 (Fujifilm Wako Pure Chemical) as per manufacturer instructions with serum diluted to 1:100 for Cre+ animal samples and 1:4 for Cre− animal samples.

### Explant preparation and culture

For the analysis of RGC degeneration, mice were culled by cervical dislocation. The eyes were immediately enucleated after death and the temporal cornea marked using a hand-held cauterizer to orient the retina during dissection. The eyes were then immediately transferred into Hanks Balanced Salt Solution (Gibco), and the retinas were rapidly dissected. They were then immediately assessed for the 0-h group or transferred to 0.4 µm polytetrafluoroethylene culture inserts (Millipore), ganglion cell layer up. The inserts were then placed into 35 mm culture dishes (Thermo Fisher) containing 1.5 mL of Neurobasal-A culture medium (Gibco) supplemented with 1% penicillin-streptomycin (Gibco), 0.8 mM L-glutamine (Gibco), 1% N2 supplement (Gibco) and 2% B27 supplement (Gibco). Explants were then cultured at 37°C, 5% CO_2_ for 72 h with the media replaced after 48 h.

Treatment groups included B Cre+ retinas and WT retinas at 0 h. For 72 h, groups included B Cre+ and WT retinas in standard media, WT retinas supplemented with 100 ng/mL *Escherichia coli* recombinant BDNF (Regeneron/Amgen partners) diluted in 1X phosphate-buffered saline (PBS), and WT retinas supplemented with a TrkB antibody agonist (ZEB85) 50 µg/mL (Zebra Biologics). Concentrations were as in Merkouris *et al.*,^6^ who had previously compared TrkB activation by ZEB85 with BDNF and NT4 and had demonstrated neuroprotective effects in the retinal explant model. Retinas from all groups were labelled using DiOlistics or prepared for RGC counts as described below.

### Optic nerve crush

Mice were anaesthetized using 5% isoflurane (Piramal Healthcare UK Ltd)/2L O_2_/min and maintained at 2.5% throughout the procedure. Topical oxybuprocaine 0.4% (Bausch & Lomb) and povidone iodine 5% (Bausch & Lomb) were applied preoperatively. Viscotears (Bausch & Lomb) was applied to the contralateral eye to prevent exposure keratopathy. An inferotemporal incision was made through the conjunctiva and Tenon’s capsule. The globe was then rotated nasally, and blunt dissection was used to expose the optic nerve. The nerve was crushed 2 mm posteriorly to the globe for 5 s using the self-closing action of Dumont N5 cross-action forceps to provide a consistent compression force across procedures. Following the nerve crush, tissues were returned to their original position, and Viscotears and a cover slip were applied to the cornea to allow direct visualization of the optic nerve head and vasculature. If the lens had become cloudy or there were any signs of compromise to the retinal vasculature from the crush, the animals were excluded. After 7 days, all mice were culled by cervical dislocation and the retinas dissected as previously described and prepared for DiOlistic labelling.

### DiOlistics and Sholl analysis

Eighty milligrams of 1.7 µm M-25 tungsten particles (Bio-Rad) were coated with 2 mg of 1-1-dioctadecyl-3,3,3,3-tetramethylindocarbocyanine (DiI) (Invitrogen) and 4 mg of 3,3-dioctadecyloxacarbocyanine perchlorate (DiO) (Invitrogen). The coated particles were then distributed on the interior surface of Tefzel tubing (Bio-rad) before the tubing was cut into 12 mm ‘bullets’.^21^ DiI-/DiO-coated tungsten particles were fired at the retinal wholemounts, ganglion cell side up, using a Helios gene gun (Bio-rad), 5 cm from the retinal surface, fired at 110 psi (helium) through a 3 µm polyethylene terephthalate membrane (Fisher) to prevent particle clumping. Explants were then incubated for 30 min in Neurobasal-A media at 37°C, 5% CO_2_ followed by fixation in 4% paraformaldehyde (PFA) for 15 min and a final nuclear stain with Hoechst (1:1000) in PBS. Finally, explants were mounted with FluorSave (Millipore) mounting media, coverslipped and allowed to dry for 1 h at room temperature.

**Figure 1:**
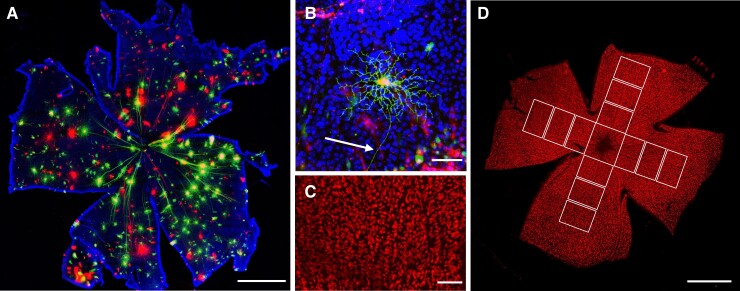
**Methods of RGC assessment.** (**A**) Flat-mounted retina labelled DiOlistically with DiI (red) and DiO (green) and Hoechst nuclear stain (blue) (scale bar = 1000 µm). (**B**) Example wild-type mouse RGC, identified by the dendritic arbour and axon (white arrow) projecting to the optic nerve (scale bar = 50 µm). (**C**) 0.33 mm^2^ sample image of wild-type cells immunolabelled for RBPMS (red) and used for cell counting (scale bar = 100 µm). (**D**) Location of sample images used to determine mean cell count per retina, taken at 500, 1000 and 1500 µm from the optic nerve head (scale bar = 1000 µm).

Flat-mounted retinas were imaged using a laser scanning microscope (LSM) 780, Carl Zeiss confocal microscope (Fig. 1A). RGCs were identified by their location in the ganglion cell layer of the retina and their morphological features, namely, a dendritic tree extending into the inner plexiform layer and an axon projecting to the optic nerve (Fig. 1B). Cells that could not be clearly delineated were excluded, either due to insufficient labelling, staining artefacts or overlapping dendritic field with neighbouring cells. Cells were imaged using ×20 objective (*z*-axis 1.67 µm) using separate channels for each dye. The (*x*, *y*) coordinates of each RGC were recorded, measured in micrometres relative to the optic nerve head.

### Neuronal reconstruction

The analysis of RGC images was conducted using Imaris software (version 9.2, Bitplane, Zurich, Switzerland) by a masked individual. Dendritic arbours were automatically reconstructed using the Filament Tracer module, with any tracing errors corrected manually. Statistical parameters were exported from Imaris and used for Sholl analysis at set intervals of 10 µm.

### RGC counts

RGCs were identified by labelling for RNA-binding protein, mRNA processing factor (RBPMS). Flat-mounted retinas, separate from those used for DiOlistic labelling, were fixed in 4% PFA before being permeabilized in 0.1% Triton in PBS for 1 h and then blocked for 1 h in 5% normal horse serum (Sigma-Aldrich) in PBS. Retinas were then incubated with primary antibody (anti-RBPMS, Novus, NBP2-20112, 1:1000 dilution) overnight at room temperature. On the following day, they were washed 3 × 10 min with PBS and then incubated in secondary antibody (goat anti-rabbit IgG, Alexa Fluor 594, Thermo Fisher R37117, 1:500 dilution) for 3 h at room temperature. After a further 3 × 10 min washes with PBS, retinas were nuclear stained with Hoechst and mounted on glass slides with FluorSave. Slides were then imaged on an LSM 780, Carl Zeiss confocal microscope.

For each retinal quadrant, 3 × 0.33 mm^2^ sample images were taken at 500, 1000 and 1500 µm from the optic nerve head (Fig. 1C and D). Cells were counted manually by a masked individual on FIJI (2.3.0/1.53f) using the Cell Counter plugin. The mean number of RGCs/image was calculated for each of the three radial distances, and a mean composite count for all three distances was calculated to produce a final cells/mm^2^ mean value for each retina.

### Statistical analysis

Animal numbers were determined using a power calculation (G*Power)^22^ of the required numbers of RGCs, based on previously reported retinal explant Sholl data^4^ and an estimate of 10 RGCs from each DiOlistically labelled retina being suitable for analysis. Statistical analyses were performed using GraphPad Prism software (version 8). Normality of data sets was tested using Shapiro–Wilk (passed normality test when alpha = 0.05). To compare two groups, unpaired *t*-test or Mann–Whitney U test were used as appropriate depending on the outcome of Shapiro–Wilk. Significant results are denoted as follows: *P < 0.05, **P < 0.01 and ***P < 0.001. For RGC dendrite analysis, a mean was calculated for each retina. A weighted mean and standard deviation (SD) were then calculated and weighted according to the number of RGCs included per retina, and statistical significance was determined by a weighted two-tailed two-sample *t*-test.^23^

## Results

### Serum BDNF concentration

**Figure 2:**
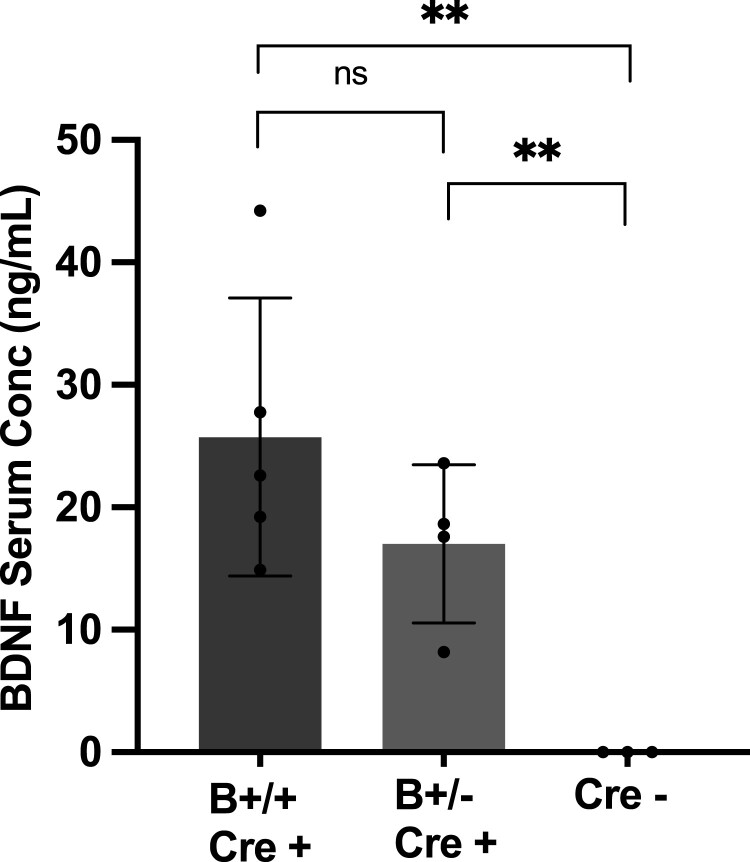
**BDNF levels in the serum of *Rosa26-LSL-Bdnf-myc-IG/Pf4iCre^+^* homozygous (B+/+ Cre+), heterozygous (B+/− Cre+) and Cre−.** *P < 0.05, **P < 0.01 and ***P < 0.001. Serum BDNF concentration measured by ELISA using the mature BDNF ELISA Kit Wako, High Sensitive, Fujifilm Wako. B+/+ Cre+ *n* = 5, B+/− Cre+ *n* = 4 and Cre− *n* = 3. Two-tailed, two-sample *t*-test B+/+ Cre+ to Cre− *P* = 0.007, B+/− Cre+ to Cre− *P* = 0.007, B+/+ Cre + to B+/− Cre + *P* = 0.194.

The concentration of serum BDNF measured by ELISA was similar to the normal range in humans, with lower levels in heterozygous compared to homozygous mice (Fig. 2). Mean BDNF concentrations were measured at 25.74 ± 11.36 ng/mL (*n* = 5) for homozygous (B+/+ Cre+) and 17.02 ± 6.44 ng/mL (*n* = 4) for heterozygous (B+/− Cre+) mice, in line with previous results using the same mouse model by Dingsdale *et al.*^17^ Using a highly sensitive and specific BDNF ELISA, very low levels of BDNF could be quantified in the serum of WT animals, 6.7 ± 2.4 pg/ml, presumably reflecting in part BDNF originating from the skeletal musculature.^24^

### RGC dendrite complexity is preserved in B+/+, Cre+ and B+/−, and Cre+ mouse retinas

**Figure 3:**
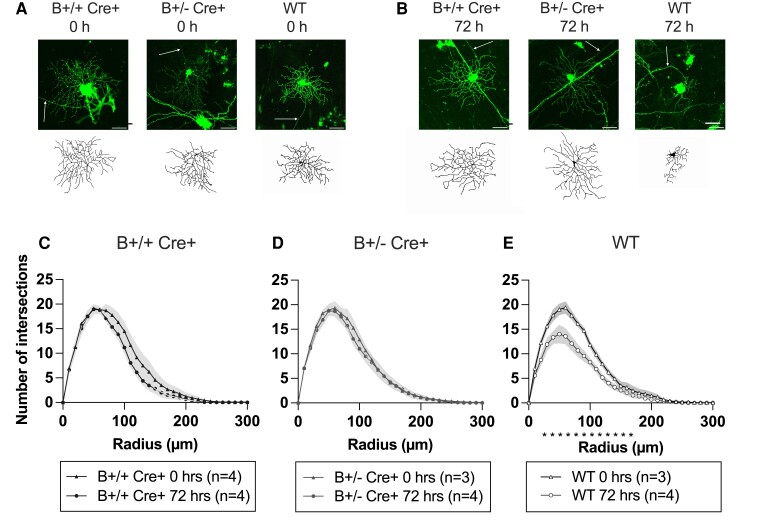
**Retinal explant results comparing RGCs from homozygous (B+/+ Cre+) heterozygous (B+/− Cre+) and wild-type (WT) retinas**. (**A**) DiOlistically labelled RGCs (DiI/DiO) and dendrite field reconstruction on Imaris in retinal explants immediately post-mortem (scale bar = 50 µm). (**B**) DiOlistically labelled RGCs (DiI/DiO) and dendrite field reconstruction on Imaris in retinal explants after 72 h. (**C–E**) Sholl analysis of reconstructed RGCs for each genotype comparing results at 0 and 72 h. Line represents weighted mean at each interval, shaded areas represent ± standard error of the mean. Weighted two-tailed, two-sample unpaired *t*-test at each interval, *P < 0.05.

**Figure 4:**
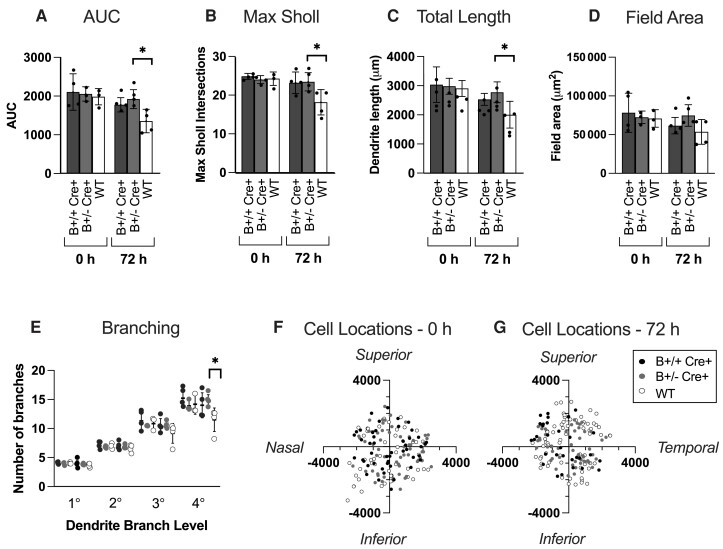
**Further analysis of retinal explant results comparing RGCs from homozygous (B+/+ Cre+) heterozygous (B+/− Cre+) and wild-type (Cre−) retinas.** (**A**) Sholl AUC, significance compared to WT at 72 h B+/+ Cre+ *P* = 0.0563 and B+/− Cre+ *P* = 0.0274. (**B**) Maximum number of Sholl intersections, significance compared to WT at 72 h B+/+ Cre+ *P* = 0.0585 and B+/− Cre+ *P* = 0.0401. (**C**) Total length of all dendrites, significance compared to WT at 72 h B+/+ Cre + *P* = 0.0799 and B+/− Cre+ *P* = 0.0379. (**D**) Dendritic field area, significance compared to WT at 72 h B+/+ Cre+ *P* = 0.04384 and B+/− Cre+ *P* = 0.0925. (**E**) Number of dendrites at each branching level proximal to the cell body, significance compared to WT at 72 h 4° B+/+ Cre + *P* = 0.144 and B+/− Cre + *P* = 0.025. For Column Scatterplots **A**–**E**, data points represent individual retinas, and columns and error bars represent weighted means and SD for group, weighted according to number of RGCs per retina. Significance was determined by weighted two-sample, two-tail *t*-test, *P < 0.05, **P < 0.01 and ***P < 0.001. (**F**, **G**) Location of cells included in analysis at 0 and 72 h, measured in micrometers relative to the centre of the optic disc.

We first determined if the B Cre+ mouse RGCs had changes in their dendritic arborization compared to WT Cre− CTLs, and if so, to determine if there was a difference between B+/+ Cre+ and B+/− Cre+ genotypes. Four mice were included for each of the B+/+ Cre+, B+/− Cre+ and Cre− WTs, both sexes, age 4–5 months. For each individual, one retina was assessed immediately, and the other was prepared for explant culture. For immediate labelling post-mortem, the dendritic arbours were similar to those seen in CTL Cre− mice (B+/+ Cre+ *n* = 4 retinas, 51 RGCs, B+/− Cre+ *n* = 3 retinas, 60 RGCs and WT Cre− *n* = 3 retinas, 51 RGCs. Four retinas were prepared for each genotype. For WT Cre− and B+/− Cre+, no cells were available from one retina due to over or under labelling). We next evaluated arbour preservation for explants maintained 72-h post-mortem. Sholl analysis demonstrated significant preservation of dendritic complexity in both genotypes compared to WT Cre− CTLs after 72 h in culture (Fig. 3) (B+/+ Cre+ *n* = 4 retinas, 31 RGCs, B+/− Cre+ *n* = 4 retinas, 49 RGCs and WT Cre− *n* = 4 retinas, 70 RGCs). The Sholl area under the curve (AUC), a global measure of dendrite complexity, was significantly larger in B+/− Cre+ (*P* = 0.0274) (Fig. 4A), with B+/+ Cre+ animals falling slightly short of significance (*P* = 0.0563) compared to WT Cre− (weighted mean AUC for B+/+ Cre+, B+/− Cre+ and WT 1775 ± 183, 1922 ± 245 and 1357 ± 304, respectively). A similar pattern was also seen with decreases in the sub-analysis of maximum Sholl intersections and total dendrite length (Fig. 4 B–D). No significant difference was seen between B+/+ Cre+ and B+/− Cre+ groups in the Sholl analysis, AUC or any of the additional sub-analyses. Based on the trends in these findings, it was considered reasonable to combine B+/+ Cre+ and B+/− Cre+ mice as a single group in subsequent experiments (referred to as B Cre+).

### Preservation of RGC dendrite complexity in B Cre+ mice is equivalent to direct BDNF media supplementation

**Figure 5:**
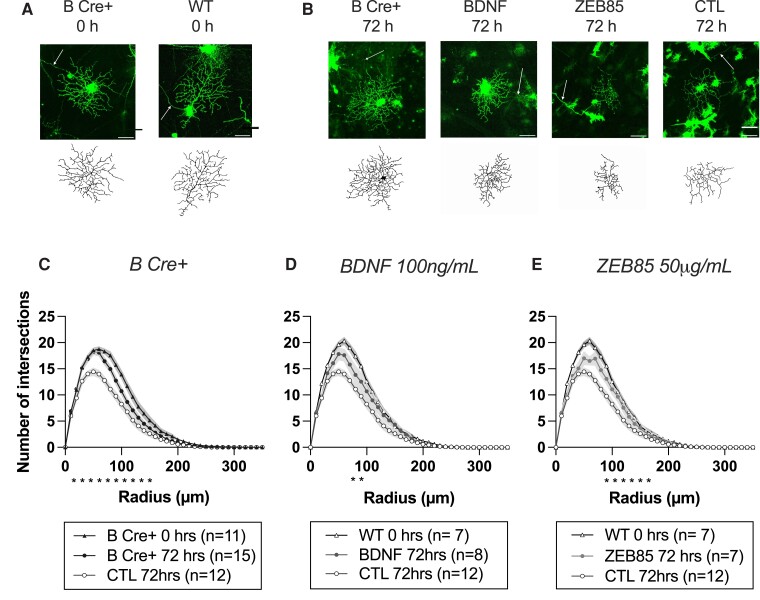
**Retinal explant results comparing *RGCs from Rosa26-LSL-Bdnf-myc-IG/Pf4iCre^+^* (homozygous and heterozygous combined, B Cre+) mice with WT retinas supplemented with BDNF, ZEB85 or CTL media.** (**A**) DiOlistically labelled RGCs (DiI/DiO) and dendrite field reconstruction on Imaris in retinal explants immediately post mortem (scale bar = 50 µm). (**B**) DiOlistically labelled RGCs (DiI/DiO) and dendrite field reconstruction on Imaris in retinal explants after 72 h. (**C–E**) Sholl analysis of reconstructed RGCs for (**C**) B Cre+ (0 h *n* = 11, 72 h *n* = 15), (**D**) BDNF media (*n* = 8) and (**E**) ZEB85 media (*n* = 7). Each Sholl plot includes the results of WT retina at 0 h (*n* = 7) and 72 h in CTL media (*n* = 12) for comparison. Line represents weighted mean at each interval, shaded areas represent ± standard error of the mean. Weighted two-tailed, two-sample unpaired *t*-test at each interval, *P < 0.05.

**Figure 6:**
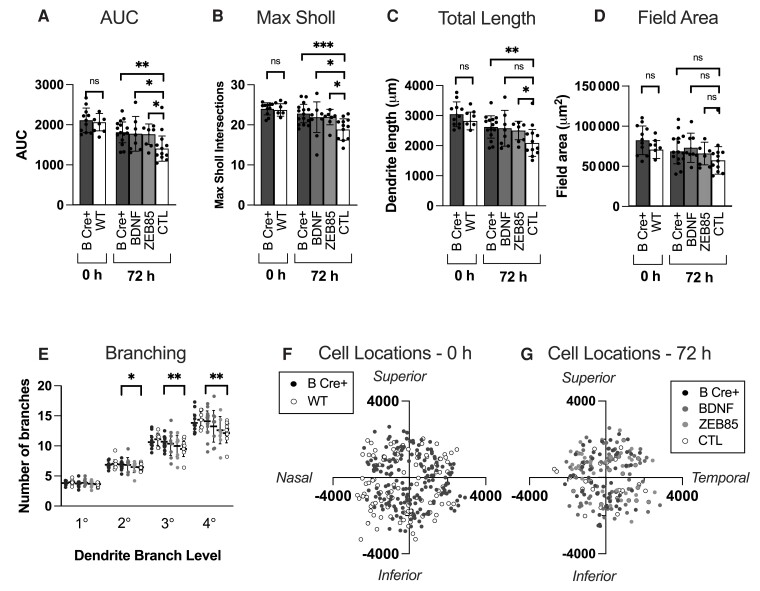
**Further analysis of retinal explant results comparing *RGCs from Rosa26-LSL-Bdnf-myc-IG/Pf4iCre^+^* (homozygous and heterozygous combined, B Cre+) mice with WT retinas supplemented with BDNF, ZEB85 or CTL media.** (**A**) Sholl AUC, significance at 72 h compared to CTL, B Cre+ *P* = 0.001, BDNF *P* = 0.040 and ZEB85 *P* = 0.0211. (**B**) Maximum number of Sholl intersections, significance at 72 h compared to CTL (B Cre+ *P* < 0.001, BDNF *P* = 0.048 and ZEB85 *P* = 0.016). (**C**) Total length of all dendrites, significance at 72 h compared to CTL (B Cre+ *P* = 0.0024, BDNF *P* = 0.0508 and ZEB85 *P* = 0.045). (**D**) Dendritic field area, significance at 72 h compared to CTL (B Cre+ *P* = 0.0879, BDNF *P* = 0.066 and ZEB85 *P* = 0.0282). (**E**) Number of dendrites at each branching level proximal to the cell body, significance at 72 h between B Cre+ and CTL (2° *P* = 0.033, 3° *P* = 0.008 and 4° *P* = 0.006. For Column Scatterplots **A**–**E**, data points represent individual retinas, and columns and error bars represent weighted means and SD for group, weighted according to number of RGCs per retina. Significance was determined by weighted two-sample, two-tail *t*-test, *P < 0.05, **P < 0.01 and ***P < 0.001. (**F**, **G**) Location of cells included in analysis at 0 and 72 h, measured in micrometers relative to the centre of the optic disc.

We next determined the relative efficacy for platelet-derived BDNF to protect RGCs compared with the external application of BDNF and a potent TrkB agonist (BDNF mimetic), ZEB85 applied to WT retinal explants. The final data (Figs. 5 and 6) included results from 28 B Cre+ retinas (12 at 0 h and 16 at 72 h) and 36 WT retinas (8 at 0 h and 12 at 72 h in CTL media, 8 at 72 h in BDNF 100 ng/mL media and 8 at 72 h in ZEB85 50 µg/mL media), both sexes, aged 2–5 months. RGCs from explants supplemented with BDNF (100 ng/mL) and ZEB85 (50 µg/mL) both showed preservation of the complexity of the dendritic field compared to the WT retinas in standard media, replicating the results of Merkouris *et al.*^6^ The retinas of B Cre+ mice in standard media also showed a level of dendrite preservation that did not differ significantly from the BDNF and ZEB85 media (B Cre+ *n* = 139 RGCs, BDNF *n* = 65 RGCs, ZEB85 *n* = 50 RGCs and WT CTL *n* = 147 RGCs) (Fig. 5). After 72 h in culture, the weighted mean Sholl AUCs were 1811 ± 258, 1776 ± 435, 1763 ± 256 and 1406 ± 315, representing a decrease from baseline of 14.3% (*P* = 0.0011), 14.0% (*P* = 0.0403) and 14.6% (*P* = 0.0211) in the B Cre+, BDNF media and ZEB85 media groups respectively, compared to 31.9% in the WT CTL group (Fig. 6A). The other morphological parameters including maximum Sholl intersections and total dendrite length showed a similar configuration to the AUC, though changes in dendrite field area did not reach significance (Fig. 6).

### RGC dendrite complexity is preserved in B Cre+ mice in following ONC

**Figure 7:**
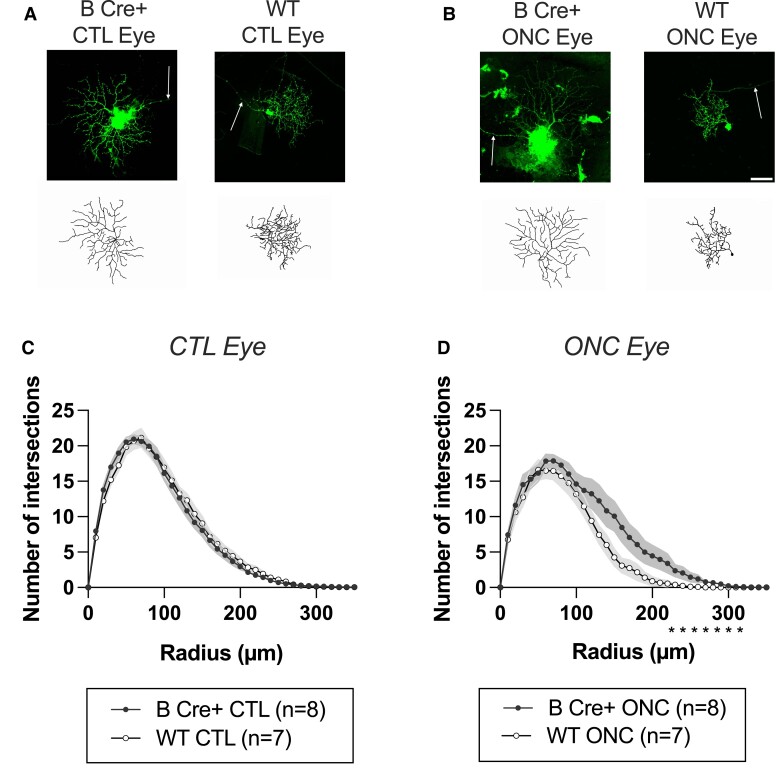
**ONC results comparing RGCs from *Rosa26-LSL-Bdnf-myc-IG/Pf4iCre^+^* (B Cre+) with WT 7 days after crush injury.** (**A**) DiOlistically labelled RGCs (DiI/DiO) and dendrite field reconstruction on Imaris in retinas from the fellow eye CTLs (CTL eye) (scale bar = 50 µm). (**B**) DiOlistically labelled RGCs (DiI/DiO) and dendrite field reconstruction on Imaris in retinas following ONC (ONC Eye). (**C**, **D**) Sholl analysis of reconstructed RGCs for (**C**) CTL eyes and (**D**) ONC eyes comparing B Cre+ mice (*n* = 8) to WT (*n* = 7). Line represents weighted mean at each interval, shaded areas represent ± standard error of the mean. Weighted two-tailed two-sample unpaired *t*-test at each interval, *P < 0.05.

**Figure 8:**
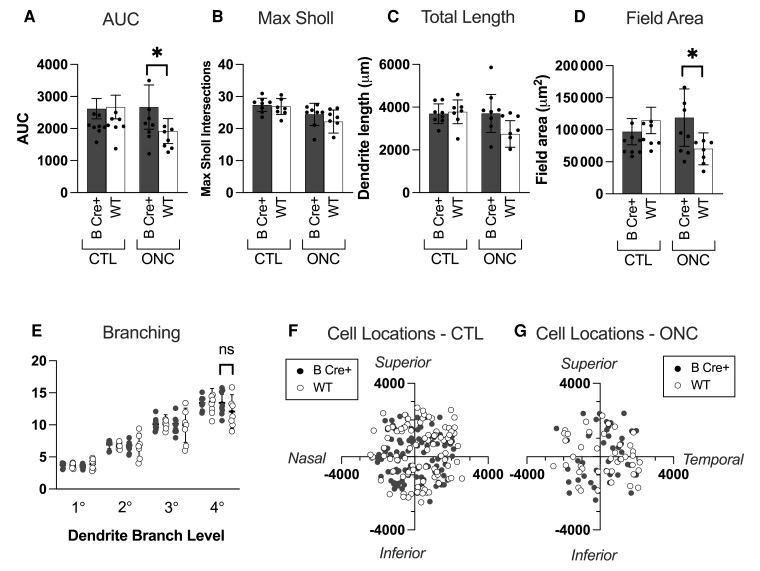
**Further analysis of ONC results comparing RGCs from *Rosa26-LSL-Bdnf-myc-IG/Pf4iCre^+^* (B Cre+) with WT 7 days after crush injury.** (**A**) Sholl AUC, ONC *P* = 0.026. (**B**) Maximum number of Sholl intersections, ONC *P* = 0.237. (**C**) Total length of all dendrites, ONC, *P* = 0.0325. (**D**) Dendritic field area, ONC *P* = 0.025. (**E**) Number of dendrites at each branching level proximal to the cell body, significance in ONC, 4° *P* = 0.2321. For Column Scatterplots **A**–**E**, data points represent individual retinas, and columns and error bars represent weighted means and SD for group, weighted according to number of RGCs per retina. Significance was determined by weighted two-sample, two-tail *t*-test, *P < 0.05, **P < 0.01 and ***P < 0.001. (**F**, **G**) Location of cells included in analysis in CTL and ONC eyes, measured in micrometres relative to the centre of the optic disc.

ONC procedures were performed on eight B Cre+ (four right eye and four left eye) mice and seven WT mice (four right eye and three left eye), both sexes, age 2–6 months (one WT mouse was culled due to bleeding during the procedure). The results from this *in vivo* ONC study demonstrated a strong neuroprotective effect on the dendrites of the RGCs in the B Cre+ mouse (B Cre+ CTL *n* = 8 retinas, 98 RGCs, WT CTL *n* = 7 retinas, 94 RGCs, B Cre+ ONC *n* = 8 retinas, 36 RGCs, and WT ONC *n* = 7 retinas, 40 RGCs) (Fig. 7). Following the ONC injury, the AUC was significantly higher in the B Cre+ mice compared to WT (2667 ± 690 and 1921 ± 392, respectively, *P* = 0.0256), with no significant difference in the contralateral eye CTLs (Fig. 8A). Total dendrite length and dendrite field area were also significantly higher (*P* = 0.0325, *P* = 0.0245). Maximum Sholl intersections failed to reach significance but continued the trend of a higher mean value in the B Cre+ group (*P* = 0.0348) (Fig. 8).

### Analysis of global RGC loss

**Figure 9:**
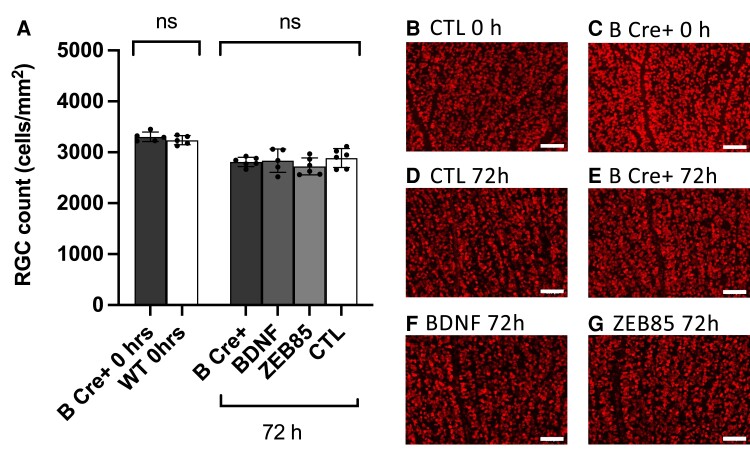
**Retinal explant results assessing RGC count using RBPMS labelling.** (**A**) Mean RGC count per retina. Data points represent individual cells, and columns represent means for group and error bars correspond to SD. Two-tailed two-sample *t*-test, 0 h *P* = 0.397, significance at 72 h compared to CTL (B Cre+ *P* = 0.397, BDNF *P* = 0.700 and ZEB85 *P* = 0.141). (**B–G**) Example images from retinal explant groups (scale bar = 100 µm) showing RBPMS positive cells (red).

**Figure 10:**
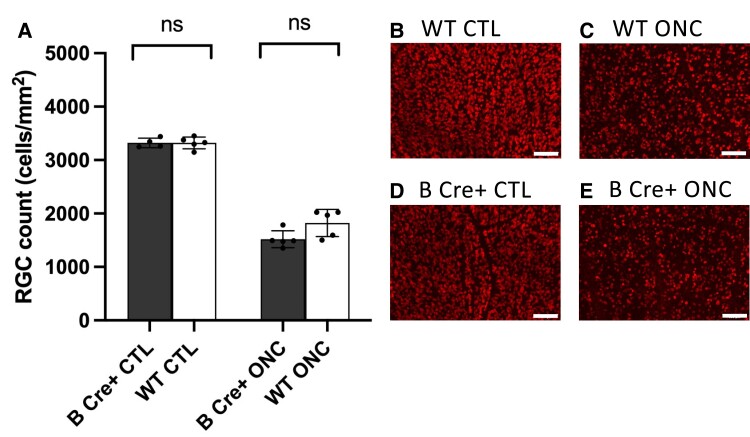
**ONC results assessing RGC count using RBPMS labelling.** (**A**) Mean RGC count per retina. Data points represent individual cells, columns represent means for group, and error bars correspond to SD. Two-tailed two-sample *t*-test CTL *P* = 0.986 and ONC *P* = 0.542. (**B–E**) Example RBPMS images from ONC experiments (scale bar = 100 µm) showing RBPMS positive cells (red).

We performed further experiments to determine the effect of platelet BDNF and exogenous BDNF on RGC survival. Retinal explants were prepared (six per group) as before [B Cre^+^ 0 h, WT 0 h, B Cre^+^ 72 h, BDNF (100 ng/mL) 72 h, ZEB85 (50 µg/mL) 72 h and CTL media 72 h], both sexes age 2–6 months. ONC was performed on a further five WT and five B Cre+ mice, both sexes, age 2.5–4 months. In contrast to the effects seen on dendrite preservation, we did not observe any difference in RGC survival between groups in either the retinal explant (Fig. 9) or the ONC experiment (Fig. 10). All retinal explants showed a reduction in RGC counts of ∼15% at 72 h with B Cre^+^, BDNF (100 ng/mL), ZEB85 (50 µg/mL) and CTL groups showing cell counts of 2811 ± 91.33, 2836 ± 229.9, 2723 ± 167.7 and 2888 ± 186.0 cells/mm^2^. In the ONC experiment, both WT and B Cre^+^ retinas showed RGC loss of ∼50% (WT 45.14% loss, B Cre^+^ 54.28% loss, not significant) 7 days after ONC, which is the observed level of RGC loss in an untreated eye. There were no differences in the RGC cell counts at baseline between the B Cre+ mice and WTs, further suggesting normal retinal development in the B Cre+ mice.

## Discussion

The main finding of this study is that dendritic atrophy of lesioned RGCs is significantly reduced by platelet BDNF, both in *ex vivo* and *in vivo* models of RGC damage. Furthermore, this reduction is similar to that achieved by direct BDNF and ZEB85 media supplementation in the retinal explant experiments. The conclusion is that the platelet BDNF exerts a significant neuroprotective effect on damaged CNS neurons. The AUC following ONC was notably higher than the AUC in the retinal explants, reflecting the more severe nature of complete axotomy compared with the crush injury. As primates contain BDNF levels similar to those determined in the engineered mouse line used here, it is conceivable that neuronal damage may be milder in primates than those observed in mice following lesions of the nervous system due to the absence of this build-in neuroprotective mechanism in mice.

While we observed a protection of dendritic arbours in surviving RGCs, the rate of RGC loss was unaffected, relative to CTLs, for all groups. Although this seems to contradict previous work showing a positive effect of BDNF on neuronal survival,^25,26^ there are important differences in study design likely to account for this apparent discrepancy. In the retinal explant model, RGC loss only becomes apparent after around 3 days,^4,19^ and previous studies did not demonstrate a neuroprotective effect of BDNF until after 4 days.^27^ Therefore, while 3 days is an appropriate duration for assessing dendrite morphology, this early time point cannot differentiate between the groups in terms of RGC survival. It does, however, highlight the strength of the DiOlistic method that allows the detection of early signs of dendritic retraction and the benefits of agents such as BDNF prior to the onset of RGC death.

The primary aim of this project was to determine changes in dendrite integrity over time as a function of the presence or absence of BDNF in platelets. The DiOlistic method used here is unsuitable to examine this question at time points later than 3 days due to the loss of RGCs and consequently the insufficient yield of labelled RGCs. The cell count was intended as a supplement to the dendritic data in order to provide a fuller description of the RGC changes at the respective time points. In future work, it would be interesting to explore potential differences in RGC counts as a function of the presence or absence of platelet BDNF at later time points following optic nerve section.

Regarding the ONC model, BDNF treatment has been shown to protect against RGC loss in this model at 7 days.^28,29^ However, it is difficult to compare dose, timing and route of delivery with the conditions used in the present study. For example, the mean serum concentration of BDNF (25.74 ng/mL) in homozygous animals is unlikely to match the much higher concentration of BDNF achieved by intravitreal injection.^29,30^ The preservation of dendrite complexity without an associated preservation of cell count is likely to represent the more subtle effects of slow, low-dose, sustained BDNF exposure in this model. Our data match the known physiological effects of BDNF in the CNS, as unlike NGF in the PNS, BDNF is not a major survival factor for most CNS neurons.^31^ Our data confirm instead the role of BDNF in supporting the integrity of the connectome, and, while platelet BDNF had a protective effect on the degenerating or retracting dendrites following axon crush or axotomy, it was insufficient to prevent the death of RGCs.

The majority of studies evaluating the therapeutic potential of administered BDNF used concentrations that are far above those found under normal physiological conditions in the CNS. This can be effective in maximally activating the TrkB pathway, but it is usually followed by its subsequent downregulation and attenuation of any neuroprotective effect. By contrast, it has been recently reported that prolonged BDNF exposure of human neurons to low BDNF concentration (1.06 ng/mL) allows TrkB phosphorylation to be reactivated following BDNF re-exposure at 24 h, unlike in the case when high initial concentrations (26.5 nM) are used.^32^ For extended use, BDNF may be most effective in repeated low doses rather than at sustained high, non-physiological concentrations. The apparently normal development of the B Cre+ mice and a neuroprotective effect on RGCs supports this interpretation. In further work, it would be of interest to determine the duration of the neuroprotective effects of low-dose BDNF in *in vivo* models of RGC damage.

The relationship between CNS and peripherally derived BDNF has been extensively discussed in the context of a range of neurological disorders, and our results raise questions regarding the mechanisms underlying the neuroprotective effects of platelet BDNF. BDNF does not cross the BBB when delivered intravenously, reflecting negligible transport across the BBB and rapid systemic clearance.^33^ Since the blood–retinal barrier (BRB) is analogous to the BBB and presents a similar obstruction to free diffusion, it would appear likely that the BRB may not be intact following lesion and that during this process, platelets release their content including BDNF in the engineered animals used here. However, it is worth considering that platelet BDNF delivery may be able to exploit other mechanisms that may allow passage of BDNF to the CNS, even through an intact barrier.

Platelets play an important role in homeostasis and are also involved in many other processes including tissue repair, inflammation, antimicrobial defence and angiogenesis.^34^ They contain secretory granules that comprise a large number bioactive molecules such as transforming growth factor-beta (TGF-beta) and platelet-derived growth factor (PDGF) that are released during the process of platelet degranulation that accompanies blood coagulation. Platelet degranulation leads to the circulatory release of BDNF whereby in humans, BDNF is carried in platelet-derived extracellular vesicles (EVs).^35^ EVs are secreted by most cells and can, in principle, deliver nucleotides, proteins and lipids to both near and distant neighbours.^36^ Resting platelets continuously produce EVs,^37^ and the number produced is greatly increased by platelet activation.^38^ The EV lipid bilayer allows them to remain stable in blood for an extended period and to penetrate key biological barriers including the BBB.^39^ As such, they have become an attractive option for drug delivery.^40^ Up to 60–90% of EVs in blood are derived from platelets,^41^ and it is conceivable that platelet EVs containing BDNF may have facilitated similar passage across an intact BBB/BRB and allowed BDNF to reach RGCs in the B Cre+ mouse. These considerations raise the question as to whether enhancing the release of BDNF in EVs through platelet activation could be applied as a clinical treatment. The storage of BDNF within platelets may decrease clearance by the kidneys and reduce the need for repeated treatments. Packaging within EVs could also solve issues arising from BDNF’s charge that prevent it reaching target tissues. Intermittent activation could allow pulses of low physiological doses of BDNF to be released in contrast to a sustained (and potentially less efficacious) elevated concentration, by reducing the downregulation of TrkB. Significant further work is required to determine parameters for the optimal stimulation of platelet activation.

In summary, we report here a significant neuroprotective effect of platelet BDNF on RGC dendrites in explants and ONC models of RGC damage. It is thus conceivable that in humans, the extent of neuronal damage observed for example after the lesion of blood vessels in the brain may be mitigated by the presence of BDNF in platelets and that lesion models in the mouse thus imperfectly reflect the situation in humans. Although BDNF on its own does not cross the BBB, platelets may also represent a functionally relevant source of BDNF and facilitate the delivery of BDNF to the CNS via EVs. Exploring methods to access this store of BDNF further could have valuable applications for the treatment of CNS disorders.

## Data Availability

RGC Imaris files containing filament tracer skeletonizations have been submitted to NeuroMorpho.org and will be available in the Want_Morgan archive. Further data supporting the findings of this study are available from the corresponding author, upon reasonable request.

## Abbreviations

ARVO =: Association for Research in Vision and Ophthalmology
AUC =: area under the curve
BBB =: blood–brain barrier
B Cre+ =: *Rosa26-LSL-Bdnf-myc-IG/Pf4iCre^+^*
BDNF =: brain-derived neurotrophic factor
*BDNF* =: human brain-derived neurotrophic factor gene
*Bdnf* =: mouse brain-derived neurotrophic factor gene
BRB =: blood–retinal barrier
Cre+ =: Cre recombinase positive
CTL =: control
DiI =: 1-1-dioctadecyl-3,3,3,3-tetramethylindocarbocyanine
DiO =: 3,3-dioctadecyloxacarbocyanine perchlorate
EV =: extracellular vesicle
GFP =: green fluorescent protein
LSL =: lox-stop-lox
LSM =: laser scanning microscope
ONC =: optic nerve crush
PDGF =: platelet-derived growth factor
PF4 =: platelet factor 4
PFA =: paraformaldehyde
PNS =: peripheral nervous system
RBPMS =: RNA-binding protein, mRNA processing factor
RGC =: retinal ganglion cell
SD =: standard deviation
TrkB =: tropomyosin receptor kinase B
WT =: wild type
ZEB85 =: tropomyosin receptor kinase B-activating antibody developed by Zebra Biologics.
